# Assessment of the alveolar volume when sampling exhaled gas at different expired volumes in the single breath diffusion test

**DOI:** 10.1186/1471-2466-7-18

**Published:** 2007-12-19

**Authors:** Renato Prediletto, Edo Fornai, Giosuè Catapano, Cristina Carli

**Affiliations:** 1 Institute of Clinical Physiology, National Research Council of Italy, Pisa, Italy

## Abstract

**Background:**

Alveolar volume measured according to the American Thoracic Society-European Respiratory Society (ATS-ERS) guidelines during the single breath diffusion test can be underestimated when there is maldistribution of ventilation. Therefore, the alveolar volume calculated by taking into account the ATS-ERS guidelines was compared to the alveolar volume measured from sequentiallly collected samples of the expired volume in two groups of individuals: COPD patients and healthy individuals. The aim of this study was to investigate the effects of the maldistribution of ventilation on the real estimate of alveolar volume and to evaluate some indicators suggestive of the presence of maldistribution of ventilation.

**Methods:**

Thirty healthy individuals and fifty patients with moderate-severe COPD were studied. The alveolar volume was measured either according to the ATS-ERS guidelines or considering the whole expired volume subdivided into five quintiles. An index reflecting the non-uniformity of the distribution of ventilation was then derived (DeltaVA/VE).

**Results:**

Significant differences were found when comparing the two measurements and the alveolar volume by quintiles appeared to have increased progressively towards residual volume in healthy individuals and much more in COPD patients. Therefore, DeltaVA/VE resulted in an abnormal increase in COPD.

**Conclusion:**

The results of our study suggest that the alveolar volume during the single breath diffusion test should be measured through the collection of a sample of expired volume which could be more representative of the overall gas composition, especially in the presence of uneven distribution of ventilation. Further studies aimed at clarifying the final effects of this way of calculating the alveolar volume on the measure of DLCO are needed. DeltaVA/VE is an index that can help assess the severity of inhomogeneity in COPD patients.

## Background

There is evidence that the gases inspired into the alveolar regions are not well mixed and that the alveolar units fill and empty sequentially [1-4]. Importantly, in the presence of prevalent lung diseases such as COPD, this process may be exaggerated because of the increased time constant of lung units [5,6]. As a matter of fact, when airways are narrowed by inflammatory cells and mucus, the distribution of the inspired gas in the alveoli becomes progressively impaired. This may result in marked differences in gas composition within the lungs, as well as in inhomogeneous patterns of filling and emptying [7,8]. The different profile of emptying and filling of the lung units causes discrepancy in their gas tracer composition, thus creating regional differences within lung units for tracer inert insoluble gases such as helium or methane [9,10]. Indeed, if there is unevenness in the distribution of a single inspiration in the dilution of the tracer gas and incomplete equilibration within respiratory units, as in the presence of airflow obstruction, the pattern of reappearance of that gas in a single expiration to residual volume will be consequently influenced [11,12]. This may account for the extreme difficulty in obtaining a sample that is representative of the overall gas composition using the most common laboratory tests [13].

It is well recognized that the single breath diffusion test [14-16] requires a measurement of the alveolar volume. According to recent ATS-ERS guidelines, such volume is based on the sampling of 750–1000 ml of expired volume after washout of the dead spaces when the tracer gases, such as helium or methane, are promptly inhaled and expected to be diluted in well-ventilated units during the manoeuvre of inspiratory vital capacity in the course of the test [17,18]. Whenever severe airflow occurs, the gas mixes less effectively and, therefore, sampling in the initial expired concentration of the gas tracer is not always correct as it is likely to overestimate the real value of alveolar gas concentrations and consequently underestimate that of the alveolar volume. This discrepancy could lead to substantial differences when calculating DLCO in COPD patients [17,19].

We hypothesize that the alveolar volume measured according to the ATS-ERS method is very different from that calculated considering subsequent phases of the expired volume in those areas where the gas composition is different owing to the fact that the slow-emptying units predominate. In the course of the single breath diffusion test, with the aid of rapid response analyzers it is now possible to follow exhalation to the residual volume after breath-hold and to measure in selected points of the exhalation process the instantaneous expired inert gas fractions which could enter into the calculations of the alveolar volume. In this way, we compared the measurements of the standard alveolar volume obtained following the ATS-ERS recommendations (VAst) to those derived by considering the whole expirate of the same single breath diffusion test, minus the dead spaces, divided into five quintiles and considering the related expired inert gas fractions (VAq) in each quintile. This procedure allowed us to evaluate whether there existed any large discrepancy between the two measurements of the alveolar volume in those cases like COPD, where the process of sequential emptying of different alveolar regions may be excessive. This comparison was made both in healthy individuals and in COPD patients.

## Methods

The study included 30 healthy individuals and 50 patients affected by COPD. The healthy subjects had no history of smoking, nor respiratory symptoms consistent with the diagnosis of COPD nor other pulmonary disease. The healthy subjects were receiving no respiratory medication nor any other medication which could affect the respiratory function. The patients with COPD fulfilled the diagnostic criteria of the Global Initiative for Chronic Obstructive Lung Disease guidelines [20]. All patients with COPD were smokers and were recruited from the pulmonary disease unit of our Institution. At the time of the study all patients with COPD were stable. Before the testing session, all subjects were asked to withhold taking inhaled short-acting β-agonist and/or anticholinergic agents. Patients had no other cardiopulmonary disease, and had experienced no upper respiratory tract infection during the previous 4 weeks. Patients with COPD were classified according to the results of spirometry as having a moderate to severe obstruction. Informed written consent was obtained for the study protocol according to the policy of our Internal Review Board. Spirometry was performed as stated by the ATS-ERS recommendations [21]. Airflow limitation was characterized by FEV_1 _and FEV_1_/VC respectively below 70% and 88% of the normal value predicted after inhalation of a bronchodilator. Bronchodilator reversibility was defined as an increase of 12% of the baseline value and 200 mL respectively for either FEV_1 _or FVC above the prebronchodilator baseline, 30 minutes after inhalation of 400 μg of salbutamol [22]. All values were expressed as percentage of reference values [23]. In order to assess the pulmonary function, we measured static lung volumes which were expressed as percentage of reference values [23]. The single breath diffusing capacity for carbon monoxide was determined using a fully computerized spirometric system (Comprehensive Pulmonary Laboratories Collins Medical Ferraris, USA-England) which was the same instrument employed for spirometry and lung volumes. The test was performed at sea level. The system was equipped with rapid infrared analyzer devices for carbon monoxide (CO) and methane (CH4). This gas analyser system provided continuous tracings of CO and tracer gas concentration during the test. DLCO was measured according to the ATS-ERS guidelines [16-18]. Each subject performed the single breath CO test in sitting position. The patients who were current smokers stopped smoking at least 24 hours before the tests. The interval between tests was only 4 minutes for healthy individuals and over 10 minutes for severe COPD patients, according to the ATS-ERS guidelines. The measured DLCO was adjusted for hemoglobin concentration. The alveolar volume was measured by dilution in the lung of 0.3% of CH4 present in the inspiratory bolus of the breath test, in the course of inspiratory vital capacity. The remainder of the test gas mixture included 0.3% of CO, 21% of oxygen and balance nitrogen.

In accordance with the purpose of this study the effective alveolar volume was calculated by two different methods. The first method directly measures tracer gas reduction during breath-holding time according to the ATS-ERS guidelines and was defined VAst (standard alveolar volume) obtained following the ERS-ATS recommendations according to the following formula:

VAst = [FICH4/FACH4(750 mL)] × [Vinsp - (Vd instrumental + VD anat)]

where FICH4 = methane concentration in the inspired gas, and FACH4 (750 mL) = methane concentration in the alveolar sample collected for 750 mL after having discarded the instrumental and anatomical dead spaces; Vinsp = inhaled volume; VD = instrumental dead space and VD anat = anatomical dead space. Sampling of the tracer gas CH4 was executed at the mouth level of the patient in real time and in correspondence of the measurement of the inspiratory and mean expiratory volumes.

**Figure 1:**
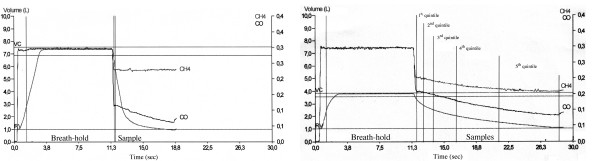
Typical tracings of CO and CH4 during exhalation of the single breath-hold CO test. An example of the standard method used to derive the alveolar volume during exhalation after the dead spaces have been entirely washed is reported for a healthy subject (left). This sample, which corresponds to the end of the knee on the CH4 and CO tracings after the hold time, usually measures between 500 and 1000 ml. An example of the method used to calculate the alveolar volume during exhalation subdividing the whole expirate into five sized quintiles in a COPD patient is reported (right). The increased slope of CO and CH4 tracings during the emptying phase when compared with the normal subject on the left is caused by the increased time-constant of emptying of lung units.

The second method, which allowed us to derive the alveolar volume subdividing the whole expirate into quintiles (VAq), uses measurements made during the same manoeuver. The difference is that the total expired space is divided into quintiles. In these quintiles the mean concentrations of CH4 were promptly read and retained for the calculations during exhalation from TLC to RV, according to the following formula:

VAq = [(FICH4/FACH4(quintile)) × (Vinsp)] where:

FACH4 (quintile) = the methane concentration in the expired volume of that quintile; Vinsp = inhaled volume. Two examples of the different ways of deriving the alveolar volume from the single breath CO manoeuver have been reported in Figure 1. From the analysis of VAq (alveolar volume measured according to the method of subdividing the whole expirate into quintiles) in our sample we derived a parameter, defined as DeltaVA/VE, which represents the changes in percentage of the alveolar volume for each litre of expired volume exhaled. This parameter was compared between healthy individuals and COPD patients.

### Statistical analysis

Group data are expressed as mean and standard deviation. The groups and functional parameters in Table 1 were compared by the unpaired Student t-test. The mean values of VAst (standard alveolar volume obtained following the ERS-ATS recommendations) and VAq (alveolar volume measured according to the method of subdividing the whole expirate into quintiles) of two consecutive tests were compared by the paired Student t-test to assess their statistical differences. Differences in mean VAst and VAq between two consecutive tests and their respective coefficients of repeatibility (defined as 2.77 × SD) were derived from healthy subjects and patients with COPD using the Bland Altman method [24]. Fisher's variance test was used to compare the repeatibilities between groups at each VAq and VAst and the level of significance was reported in Table 2. Frequency distribution of changes in percentage of DeltaVA/VE for each litre of expired volume exhaled was employed in our two samples and was graphed as box-wisker plot diagram where the horizontal lines in the box represent the 50th percentile median, the borders of the box the 25th and 75th percentiles of distribution and the wiskers the 10th and 90th percentiles.

**Table 1:** Demographic and functional characteristics of the study population

		"Healthy" individuals (n = 30)	COPD patients (n = 50)	Significance
Age	years	65 ± 6	68 ± 6	ns
Weight	Kg	71 ± 10.48	75 ± 12.53	ns
Height	cm	162 ± 9.58	168 ± 6.44	p < .0038
BMI	Kg/m^2^	27 ± 4	27 ± 6	ns
Pack-years	n	0	43.4 ± 17.5	NA*
Males	%	47	92	NA*
Hemoglobin	g/dl	13.97 ± 1.07	14.55 ± 1.07	p < .02
FVC	%pred	120 ± 13	84 ± 17	p < .0001
FEV_1_	%pred	116 ± 15	45 ± 12	p < .0001
FEV_1_/VC	%pred	97 ± 7	53 ± 13	p < .0001
VC	%pred	119 ± 13	87 ± 16	p < .0001
RV	%pred	102 ± 22	145 ± 37	p < .0001
TLC	%pred	106 ± 11	106 ± 14	ns
RV/TLC	%pred	95 ± 15	131 ± 22	p < .0001
DLCO	%pred	95 ± 17	64 ± 22	p < .0001
VA	%pred	93 ± 10	80 ± 13	p < .0001
DL/VA	%pred	97 ± 14	71 ± 26	p < .0001

Demographic and functional characteristics of the study population.

Data are expressed as mean ± standard deviation. Significant differences appear at the 0.05 level.

Abbreviations and references for the predicted formulas of each variable can be seen in the text. * Not applicable

**Table 2:** Coefficients of repeatability (defined as 2.77 × SD) for VAst (standardalveolar volume obtained following the ERS-ATS recommendations) and VAq (alveolar volume measured according to the method of subdividing the wholeexpirate into quintiles) between two consecutive tests for healthy individuals and patients with COPD using the method of Bland-Altman (24) and comparison of the repeatibilities between groups (healthy individuals and COPD patients)

	Healthy individuals (n = 30)		COPD patients (n = 50)		
		°p <		*p <	**p <

VAst	± 144 ml	.073	± 165 ml	.011	.753
VAq (first)	± 78 ml	.481	± 58 ml	.198	.999
VAq (2nd)	± 71 ml	.745	± 86 ml	.022	.843
VAq (3rd)	± 80 ml	.825	± 129 ml	.161	.992
VAq(4th)	± 95 ml	.356	± 78 ml	.256	.149
VAq (5th)	± 80 ml	.740	± 94 ml	.507	.787

Coefficients of repeatability (defined as 2.77 × SD) for VAst (standard alveolar volume obtained following the ERS-ATS recommendations) and VAq (alveolar volume measured according to the method of subdividing the whole expirate into quintiles) for healthy individuals and patients with COPD using the Bland-Altman method (24), in order to examine the variation between two consecutive tests. The level of statistical significance in terms of probability (p) has been reported. It is evident that the variation in the calculated alveolar volume between two tests appeared statistically significant for the VAst of COPD patients. In the healthy individuals it resulted approximately close to the level of significance. On the contrary, for the alveolar volumes calculated by the quintile method, the variation between two consecutive tests was only significant in the 2nd quintile of the COPD patients. In addition, no differences were observed in the comparison in repeatibilities between groups at each VAq (and VAst).

level of significance related to the comparison between coefficients of repeatibilities within two measurements for healthy individuals (°p < ) and COPD patients (*p < );

level of significance of the repeatibilities by the analysis of variance between groups (healthy individuals and COPD patients) (** p < )

## Results

### Subjects' characteristics

Demographic characteristics and parameters of lung function in healthy subjects and COPD patients have been reported in Table 1. Our COPD patients were different only in terms of height when compared to healthy individuals who had a history of smoking; males were more present in the COPD group when compared to healthy individuals (92% vs 47% respectively) and only 8% of 50 COPD patients were females, whereas the control group was close to half female, indicating a disproportion between males and females in this cohort of patients and control group which surely indicates a gender difference in terms of smoking habit. Functional parameters showed signs of severe airflow obstruction and of hyperinflation in COPD patients. DLCO single breath was significantly reduced in COPD patients, as well as its ratio to alveolar volume. With regard to patient performance of the single breath test, 3 healthy individuals and 7 COPD patients were discarded because their inspiratory time during the manoeuver was over 4 seconds, while 9 healthy subjects and 34 COPD patients were discarded because their breath-hold time was too long. The dead space washout volumes (including instrumental, filtering and anatomic dead space) resulted 563 ± 134 ml (range 150–750 ml) and 447 ± 134 (range 340–750 ml) respectively in normals and in patients with COPD. The sample collection volumes were 561 ± 195 ml (range 240–920 ml) and 490 ± 140 ml (range 240–880 ml) respectively in healthy individuals and in patients with COPD.

### Calculated alveolar volume, choice of quintiles and repeatibility between tests

The ideal approach would be to compute automatically, point by point, as a continuum, the FICH4/FECH4 ratio during the whole expiration in the course of the single-breath CO test. In order to evaluate whether the sampling of five quintiles was sufficient, we subdivided the whole expired volume into a series of four, five, six and ten exact portions. The alveolar volume calculated using four portions was significantly different when compared to that obtained using five, six or ten portions in both healthy individuals and in COPD patients. At the same time, no differences were observed when the calculated alveolar volumes, obtained subdividing the whole expirate into six portions, were compared to five or ten exact portions both in healthy individuals and in COPD patients. As a result of this prior analysis, the method of sampling five quintiles appeared sufficiently good, more precise than the use of four portions, not different from the use of six or ten portions. Therefore, the method was considered suitable for the purpose of our study and was ultimately chosen and compared to the ERS-ATS method of measuring the alveolar volume.

The repeatability of VAst (standard alveolar volume obtained following the ERS-ATS recommendations) and of VAq (alveolar volume measured according to the method of subdividing the whole expirate into quintiles) was tested both in healthy individuals and in patients with COPD and has been reported in Table 2.

It is evident that variation in the calculated alveolar volume between two tests appeared statistically significant for the VAst of COPD patients. In the healthy individuals it resulted approximately close to the level of significance. On the contrary, for the alveolar volume calculated by the quintile method, the variation was only statistically significant for the 2nd quintile of the COPD patients. No other significant variations were observed in the healthy individuals nor in the other quintiles of the COPD patients. In addition, the healthy individuals and COPD patients were not different in repeatability (Table 2).

**Figure 2:**
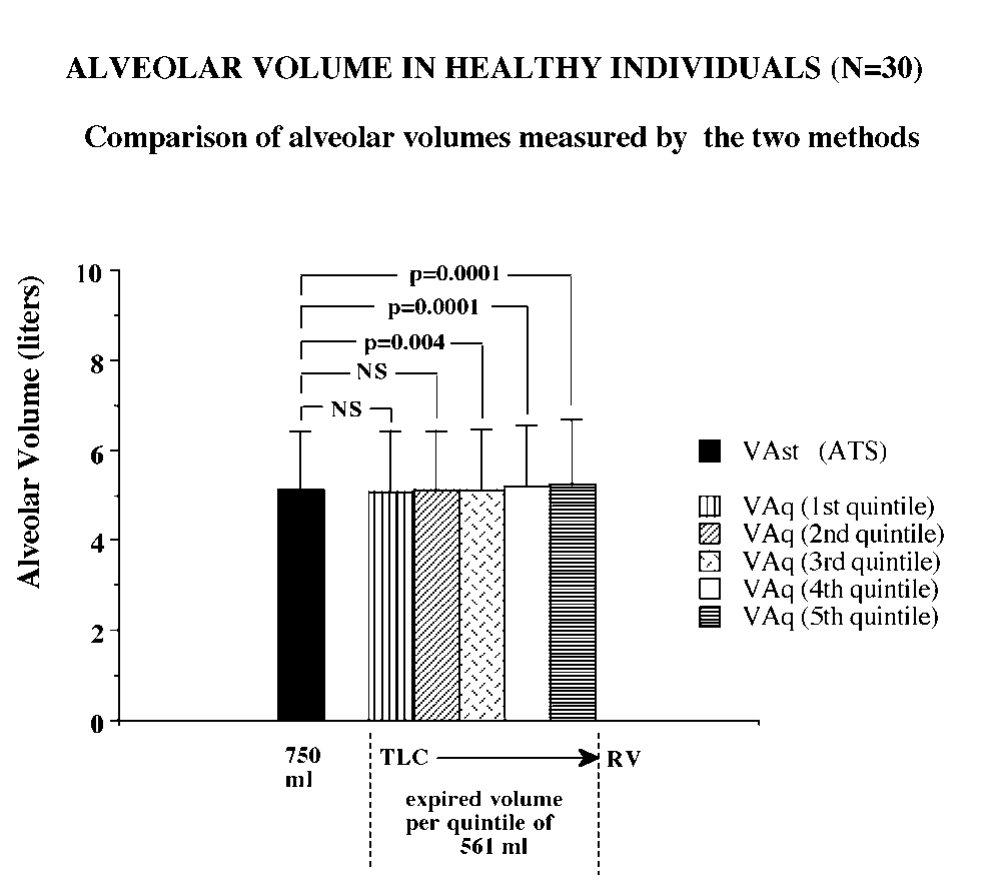
Graphic representation of the alveolar volume calculated by the two methods in healthy individuals. The bars represent the mean values and the lines above the bars represent one standard deviation from the mean values. The alveolar volume calculated by the quintile method appears significantly different from that calculated by the ERS-ATS standard (the left hand image is taken from reference 18) from the third quintile, corresponding to 40% of exhaled volume, to residual volume (RV) from total lung capacity (TLC).

**Figure 3:**
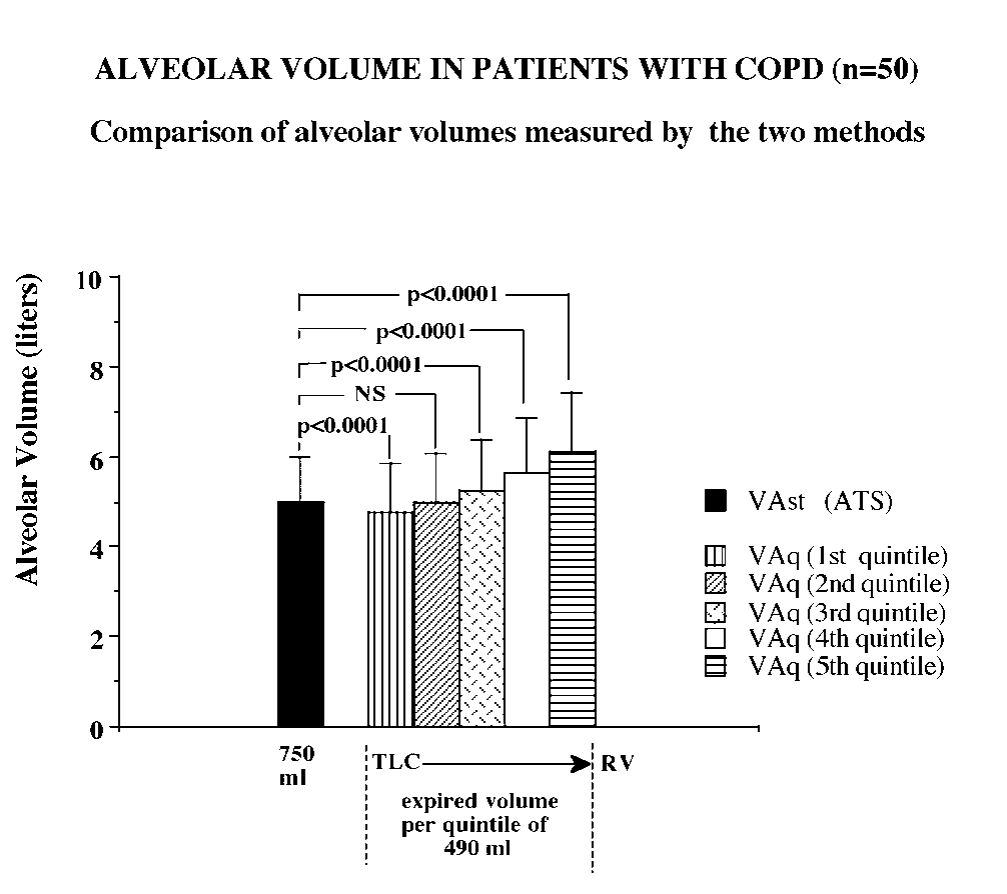
Graphic representation of the alveolar volume calculated by the two methods in COPD patients. The bars represent the mean values and the lines above the bars represent one standard deviation from the mean values. The alveolar volume calculated by the quintile method appears significantly different from that calculated using the standard method for all quintiles, except for the second one. It is evident that the alveolar volume, measured on the instantaneous CH4 fraction of each quintile, progressively increases from the beginning to the end of exhalation from total lung capacity (TLC) to residual volume (RV).

**Figure 4:**
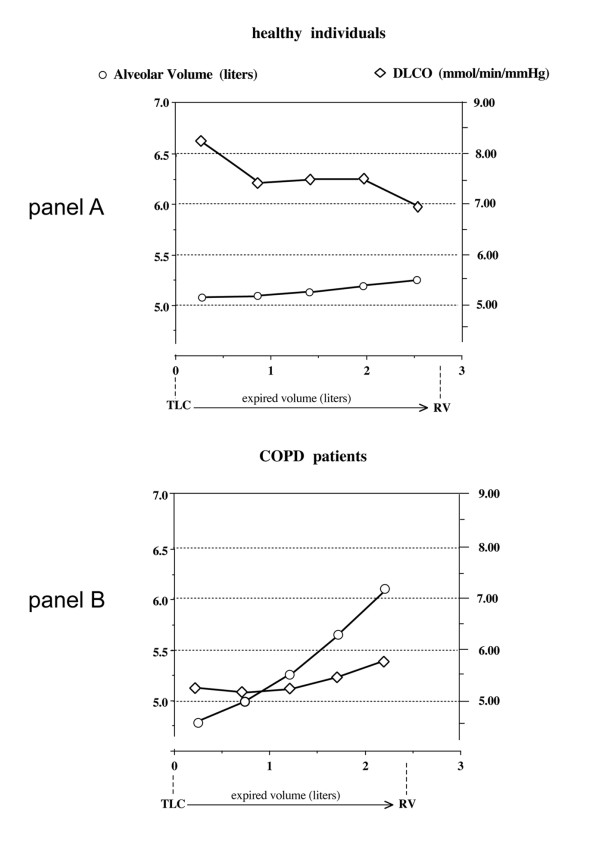
Graphic representation of the relation between the mean values of alveolar volume, calculated by the quintile method, and those of the corresponding DLCO, in healthy individuals (panel A) and in COPD patients (panel B), at different expired volumes. The alveolar volume does not show any remarkable change when related to the expired volume in healthy individuals (only 300 ml), at variance with those of COPD patients. In addition, DLCO decreases by 1.5 mmol/min/mmHg with respect to the slight changes of the alveolar volume in healthy individuals, whereas it increases by less than 1 mmol/min/mmHg for a total increase of 2.5 litres of alveolar volume in COPD patients from TLC to RV.

The results of the comparison between the two ways of measuring the alveolar volume have been reported in Figures 2 and 3, for healthy individuals and COPD patients respectively. In the healthy individuals the values of the alveolar volume were not significantly different between the two methods of assessment in the first 20 and 40% of exhaled volume. Significant differences were instead detected in the following portions of expired volume when exhaled to residual volume. In the patients with airflow obstruction there were remarkable differences between the two methods of measuring the alveolar volume at the beginning of exhalation and were present throughout the emptying phase to residual volume. The alveolar volume measured in the last quintile appeared significantly greater than that measured in the previous quintiles, thus suggesting an effect related to the sequential emptying of lung units. As reported in Figure 4, it is evident that the alveolar volume does not exhibit any remarkable changes when related to the expired volume in healthy individuals at variance with those of the COPD patients. This result suggests a progressive increase of alveolar volume along with the process of emptying of the lung units in patients with disease.

**Figure 5:**
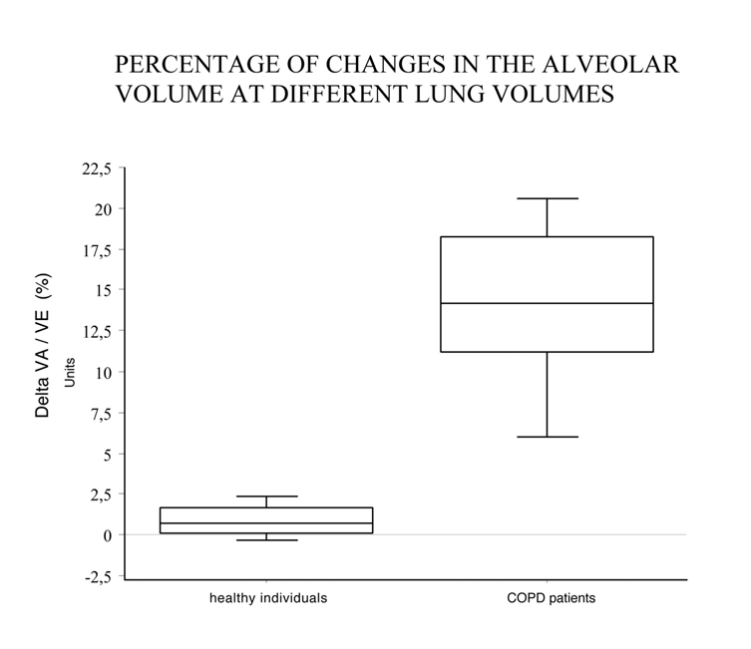
Box-wisker plots of the percentage changes of VA per litre of expired volume (Delta VA/VE) in healthy individuals and in COPD patients. The horizontal lines represent the 50th percentile (median); limits of boxes are the 25th and 75th percentiles; the wiskers are the 10th and 90th percentiles. More than 90% of patients with COPD showed significant changes in alveolar volume when sampled at different intervals of lung volume. This suggests a different time constant of lung units coupled with a non-homogeneous distribution of ventilation.

The frequency distribution of DeltaVA/VE (changes in percentage of the alveolar volume for each litre of expired volume exhaled) at different lung volumes was compared between healthy individuals and COPD patients in the box-plot graph of Figure 5. Ninety percent of COPD patients showed a progressive increase of the alveolar volume along with exhalation which was approximately more than 20% with respect to normals. Thus, it appears that the changes of alveolar volume during exhalation are quite relevant in COPD patients and that uneven emptying is necessary for these results, but not sufficient; uneven dilution ratios are also necessary.

## Discussion

The main findings of this study are as follows: 1. the measure of the alveolar volume is different depending on the point where sampling for evaluation of the alveolar concentration of inert tracer gas methane is done in the course of exhalation of the single breath diffusion test; 2. the alveolar volume measured by the quintile method shows a progressive increase from total lung capacity to residual volume and appears significantly different when compared with that measured according to the ATS-ERS guidelines; 3. its size increases much more in COPD patients than in healthy individuals from the beginning to the end of the exhalation; 4. changes of the mean alveolar volume per litre of the expired volume exhaled, expressed as DeltaVA/VE, are significantly and remarkably greater in COPD patients with severe airflow obstruction than in healthy individuals; 5. DeltaVA/VE represents a parameter that is influenced by the effect of non-uniform distribution of convective ventilation as well as by the increased time-constants of the emptying of lung units in diseased lungs.

It is well documented that the single breath diffusion test may markedly increase or slightly change upon the effect of variation in lung volume [14,15,25-29].

Although this study did not provide any conclusive information on how the final DLCO can be affected by the changes of lung volume as well as by size and precise estimate of the alveolar volume, in our COPD patients it seems to increase on the average by 1 mmol/min/mmHg for a total increase of 2.5 litres of alveolar volume, as reported in Figure 4. A different type of behaviour was observed in the healthy individuals who showed values of DLCO that decreased by 1.5 mmol/min/mmhg when compared to the slight changes of the alveolar volumes.

The purpose of our study was to indicate the weakness of the ATS-ERS method to measure the alveolar volume, which collects the alveolar inert gas concentration at the beginning of exhalation, especially in diseased lungs. In fact at this exact point (on the average a volume of 750 ml) the concentration of the tracer gas is not representative of the mean alveolar gas concentration and thus of the real alveolar volume, since it represents only the behaviour of the faster lung units [11,17]. This feature is remarkable in patients with severe airflow obstruction and is less evident in healthy individuals [30]. We could argue that in subjects like our healthy individuals, who are non smokers, and whose lungs have a near-normal distribution of ventilation, sampling of the alveolar inert gas CH4 at the beginning of exhalation does not appear much influenced by the effect of differences in the physiological regional dilution during inhalation of the test gas [7,31]. Instead, in the case of our patients affected by airflow obstruction and with signs of hyperinflation of their lungs, extreme non-uniformity of ventilation may predominate.

Literature well documents the extent to which the distribution of ventilation becomes progressively more inhomogeneous at high lung volume [32] or under the effect of modifications in tidal volumes, flows, posture and in the presence of asymmetrical geometry of lung units as well as in normal lungs [31,33]. In this study, no striking differences were detected when all healthy individuals and COPD patients were reclassified according to their significantly higher or lower expired flows and consequently tested to search any relations with the calculated alveolar volumes. This analysis showed only a slight increase of VAst (standard alveolar volume obtained following the ATS-ERS recommendations) when it was associated with a higher flow, not exhibited by the calculated VAq (alveolar volume measured according to the method of subdividing the whole expirate into quintiles). This result indicates that VAst (standard alveolar volume obtained following the ATS-ERS recommendations) is more sensitive to the different profile of emptying of alveoli when compared to the method of quintiles, which always takes into account its standardization for the whole expired volume of the subject (which is 20% of the whole expirate).

The exaggerated asymmetry of lung units caused by the obstructive airway diseases may ultimately be responsible for the inequality of gas concentration within alveolar gas and, therefore, for the very inhomogeneous dilution of the concentration of the tracer gas methane in the course of the single breath CO inhalation test.

In 1978 Ferris *et al.*[34] compared the single breath helium dilution alveolar volume and a 7-min rebreathing helium alveolar volume in normals and in COPD patients, and found that the alveolar volume measured according to the two different methods did not underestimate DLCO in the population of normals except for those patients with severe airflow obstruction. In conclusion, the authors suggested that the single breath method may be adequate for normals, but not for COPD, since the variations and fluctuations in the alveolar gas concentrations during exhalation are responsible for an incorrect estimate of the alveolar volume. This led the authors to conclude that more accurate methods for the alveolar volume were required [17,35].

Some explanations for the remarkable differences in the values of the alveolar volume when measured at the different intervals of exhalation are well reported in the paper by Yuh T Huang *et al.*[36]. These authors provided strong evidence in the evaluation of the volume-dependent distribution of DLCO in normals at rest and during exercise; they showed that the intrabreath DLCO during single exhalation in the healthy subjects was non linear and could be described by a polynomial model. The two explanations for such behaviour seem to be in accordance with the results found in our study: one explanation entails the sequential emptying profile of the lung (i.e. the " first in-last out phenomenon") which accounts for the difference in the contribution of alveolar gas concentration. Indeed, it is well known that the gas sampled at higher lung volume generally reflects the contribution from the lower part of the lung and of the faster units, which empty earlier and probably have a different regional concentration of tracer gas. The other explanation may reside in the time-constants of the lung units, which are increased in diseased lungs. As a matter of fact, by looking at the methane profile during exhalation of a COPD patient (Figure 1, right side) its steeper decreasing slope from TLC to RV is evident. This increase may primarily suggest that the recovery of the concentration of the inhaled inert gas is progressively diminishing towards residual volume, which may result in an increased time constant of lung units. The heterogeneous profile of inert gas methane during exhalation, amplified in COPD, is ultimately responsible for the differences in the calculations of the alveolar volume and it allowed us to derive the index named DeltaVA/VE (changes in percentage of the alveolar volume for each litre of expired volume exhaled), which seems to reflect the effect of the phenomena described above. In fact, this index DeltaVA/VE, as direct expression of the non-uniform distribution of ventilation, resulted significantly higher – in quantitative terms – in COPD than in healthy individuals (Figure 5). As a matter of fact, we propose this index as a very rapid, non invasive and simple tool that can be obtained routinely in patients to help the pulmonologists evaluate the effect of inhomogeneity of ventilation in the course of the single breath-hold test. Finally, the changes of the alveolar volume from TLC to RV measured by quintiles were 0.110 litres in the healthy individuals, 1.332 litres in COPD patients (Figures 2 and 3). Instead, when we use the ATS-ERS method to compare the alveolar volume with that derived from the average of the alveolar volume measured in each quintile we find similarity in healthy individuals (VAst 5.10 ± 1.33 litres vs VAq 5.15 ± 1.34 litres, p = ns) but significant differences, as expected, in COPD patients (VAst 4.98 ± 1.04 vs VAq 5.36 ± 1.57 litres, p < 0.0001).

It follows that the true mean alveolar volume should be that derived from the average of each alveolar volume exhaled in each quintile (Figures 2, 3).

## Conclusion

In summary, our study provides additional information on the real estimate of the alveolar volume when different sampling points are used in the course of the single breath diffusion test for the assessment of diffusing capacity. A model was developed which subdivided into 5 parts the total volume of air exhaled after the breath-hold manoeuver was developed. The instantaneous concentration of tracer gas methane was considered in each quintile; the calculation of the alveolar volume was consequently derived and compared with that derived from the traditional method according to the ATS-ERS recommendations. A significant difference was found between these two ways of measuring the alveolar volume, and the results showed significant differences in COPD patients. The conclusion drawn is that sampling at the beginning of exhalation of the single breath-test is not representative of the real mean alveolar gas concentration, especially when an important ventilation/perfusion mismatch is present. A non-uniform distribution of ventilation, coupled with an exaggerated time constant of emptying of lung units, seems ultimately to be the mechanism responsible for the differences in the size of the alveolar volume when measured differently in the course of expiration. An index reflecting this process was identified which appeared useful to assess the degree of non-uniformity of the ventilation distribution. These analyses provide a basis for further study in order to test the effects on DLCO of this way of measuring the alveolar volume from the sampling of the instantaneous tracer gas concentrations at different intervals of exhalation, but also to observe the behaviour of the diffusivity of carbon monoxide at different intervals of exhalation, as a direct consequence of the complex emptying process of lung units in different diseased states.

## Abbreviations

DLCO = single breath diffusion capacity for carbon monoxide; VA = alveolar volume; VAst = alveolar volume measured according to the ERS-ATS guidelines; VAq = alveolar volume measured according to the method of subdividing the whole expirate into quintiles and considering the instantaneous expired tracer gas fraction in each quintile; FEV_1 _= forced expiratory volume in one second; FEV_1_/VC: ratio of forced expiratory volume in one second to vital capacity or Tiffeneau index; FVC = forced vital capacity; TLC = total lung capacity; RV = residual volume; FRC = functional residual capacity; RV/TLC = ratio of residual volume to total lung capacity; BMI = body mass index expressed in Kg/m^2^; VD anat = anatomical dead space; VD instrumental = instrumental dead space; VI = inspiratory volume; Hb= concentration of hemoglobin expressed as g/dl of blood; FICH4 = inspiratory fraction of tracer gas methane; FACH4= alveolar fraction of tracer gas methane; DeltaVA/VE = changes in percentage of the alveolar volume for each litre of expired volume exhaled; ATS = American Thoracic Society; ERS = European Respiratory Society; SD = standard deviation.

## Competing interests

The author(s) declare that they have no competing interests.

## Authors' contributions

RP participated in the design of the study and drafted the manuscript, EF and GC participated in the design of the study and helped to perform it, CC participated in the design of the study and performed the functional tests. All authors read and approved the final manuscript.

## Pre-publication history

The pre-publication history for this paper can be accessed here:


